# Feasibility and outcomes of robot-assisted partial splenectomy for benign splenic lesions: a single-center experience

**DOI:** 10.3389/fsurg.2025.1726163

**Published:** 2026-01-12

**Authors:** Xi Chen, Ting Jiang, Yonghai Peng, Ruizi Shi, Chuan Qin, Hua Luo, Xintao Zeng, Pei Yang, Jianjun Wang

**Affiliations:** Department of Hepatobiliary Surgery, Mianyang Central Hospital, School of Medicine, University of Electronic Science and Technology of China, Mianyang, China

**Keywords:** benign splenic lesion, partial splenectomy, robot-assisted surgery, spleen preservation, three-dimensional reconstruction

## Abstract

**Background:**

To evaluate the feasibility, safety, and short-term clinical outcomes of robot-assisted partial splenectomy (RAPS) in the treatment of benign splenic lesions (BSLs).

**Methods:**

A retrospective analysis was conducted on nine patients with BSLs who underwent RAPS in the Department of Hepatobiliary, Pancreatic, and Splenic Surgery at Mianyang Central Hospital between January 2024 and September 2025. Clinical data, including demographic characteristics, lesion features, intraoperative parameters, postoperative recovery, and complications, were collected. All patients underwent preoperative contrast-enhanced abdominal computed tomography, magnetic resonance imaging, and three-dimensional (3D) reconstruction to delineate the anatomical relationship between the lesion and splenic vasculature. All procedures were performed by the same surgical team.

**Results:**

All nine procedures were successfully completed without conversion to open surgery. The cohort comprised three men and six women, with a mean age of 49.0 ± 10.3 years. Lesions were located in the lower pole in seven cases and in the upper pole in two, with a mean diameter of 4.34 ± 0.8 cm. The mean operative time was 179.4 ± 15.5 min, the mean intraoperative blood loss was 71.1 ± 19.6 mL, and the mean postoperative hospital stay was 6.4 ± 0.9 days. No cases of splenic infarction, pancreatic fistula, hemorrhage, or severe infection were observed. Pathological diagnoses included splenic hemangioma (*n* = 3), non-parasitic splenic cyst (*n* = 5), and splenic lymphangioma (*n* = 1). During follow-up, no recurrence, new lesions, or splenic dysfunction were detected, and hematologic parameters remained within normal ranges.

**Conclusion:**

RAPS is a safe, feasible, and minimally invasive spleen-preserving procedure. Preoperative 3D reconstruction facilitates precise surgical planning, and when combined with the high-precision maneuverability of robotic technology, enables complete lesion removal while preserving functional splenic tissue. This approach aligns with the principles of modern precision and minimally invasive surgery.

## 1 Introduction

The spleen plays important roles in immune regulation and hematologic homeostasis, although it is not indispensable for survival (1–3). Historically, due to limited understanding of splenic function, total splenectomy was widely adopted as the standard surgical treatment for splenic space-occupying lesions (4). Although total splenectomy can achieve complete lesion removal, postoperative complications such as impaired immune function, thrombocytosis, and overwhelming infection have increasingly drawn clinical attention (5).

With the advancement of minimally invasive surgery and a growing emphasis on spleen preservation, partial splenectomy (PS) has gradually become the preferred approach for managing benign splenic lesions (BSLs). The primary goal of PS is to achieve radical removal of the lesion while preserving adequate functional splenic tissue to maintain immune competence and reduce postoperative complications (6, 7).

However, the spleen's rich vascularity, friable parenchyma, and intricate hilar anatomy pose substantial technical challenges during laparoscopic partial splenectomy (LPS). In particular, when lesions are located near the splenic hilum or in cases with vascular variations, the restricted operative space, difficulty in delineating the transection plane, and the need for delicate hemostasis of the splenic parenchyma as well as precise suturing when small vascular branches are injured significantly increase the risk of intraoperative bleeding, thereby limiting the wider adoption of LPS (6).

The advent of robot-assisted surgical systems has introduced a promising technological alternative for spleen-preserving procedures. The robotic platform provides a high-definition three-dimensional (3D) view, enhanced magnification, and articulated multi-degree-of-freedom instruments, enabling meticulous dissection and precise suturing even within narrow anatomical spaces. These advantages significantly enhance surgical precision and safety. Previous reports have demonstrated that robot-assisted partial splenectomy (RAPS) is both feasible and reliable for managing complex hilar vasculature and lesions located in the splenic poles (8, 9).

Nevertheless, systematic clinical investigations of RAPS for BSLs remain scarce worldwide, and large-sample case series are particularly lacking. This study retrospectively analyzed clinical data from patients with BSLs who underwent RAPS at our institution since 2024, aiming to evaluate its feasibility, safety, and short-term outcomes, and to provide a reference for the further clinical standardization and application of this surgical technique.

## 2 Materials and methods

### 2.1 General information

This study was designed as a retrospective case series. A total of nine patients who underwent RAPS in the Department of Hepatobiliary, Pancreatic, and Splenic Surgery at Mianyang Central Hospital between January 2024 and September 2025 were included. All patients were preoperatively diagnosed with localized BSLs by contrast-enhanced abdominal computed tomography (CT) or magnetic resonance imaging (MRI). Surgical indications were confirmed through a multidisciplinary team discussion. Patients with malignant tumors or severe systemic disease were excluded.

Inclusion criteria included: (1) Imaging findings consistent with focal BSLs located in the splenic pole or peripheral segment, with suitable anatomy for partial resection; (2) Clear intraoperative demarcation between the lesion and normal splenic tissue, allowing preservation of sufficient splenic perfusion; (3) Absence of severe coagulopathy, cardiopulmonary dysfunction, or other contraindications to general anesthesia and pneumoperitoneum.

Exclusion criteria included: (1) Imaging or intraoperative suspicion of malignancy; (2) History of major upper abdominal surgery resulting in dense hilar adhesions; (3) Active systemic infection, bleeding diathesis, or other surgical contraindications.

The study protocol was approved by the Ethics Committee of Mianyang Central Hospital (Approval No. 2023HYX032), and written informed consent was obtained from all patients.

### 2.2 Preoperative evaluation and preparation

All patients underwent both contrast-enhanced abdominal CT and contrast-enhanced MRI as part of the routine preoperative evaluation. CT was used to assess lesion morphology, size, and vascular anatomy, while MRI provided complementary soft-tissue contrast and improved characterization of cystic or vascular lesions when CT findings were inconclusive. Three-dimensional reconstruction was performed using Xingyuan International Imaging Reporting System (Xingyuan International Imaging Technology Co., Ltd., China).

Routine preoperative evaluations included complete blood count, coagulation profile, liver and renal function tests, and immunologic assessment, including serum immunoglobulin levels (IgG, IgA, IgM) and lymphocyte subset analysis (CD3⁺, CD4⁺, CD8⁺ T cells). When necessary, splenic artery computed tomography angiography was performed in patients with unclear arterial branching patterns or atypical vascular variations on initial CT/MRI to confirm the 3D reconstruction findings and ensure intraoperative vascular safety.

### 2.3 Robotic system and patient positioning

**Figure 1:**
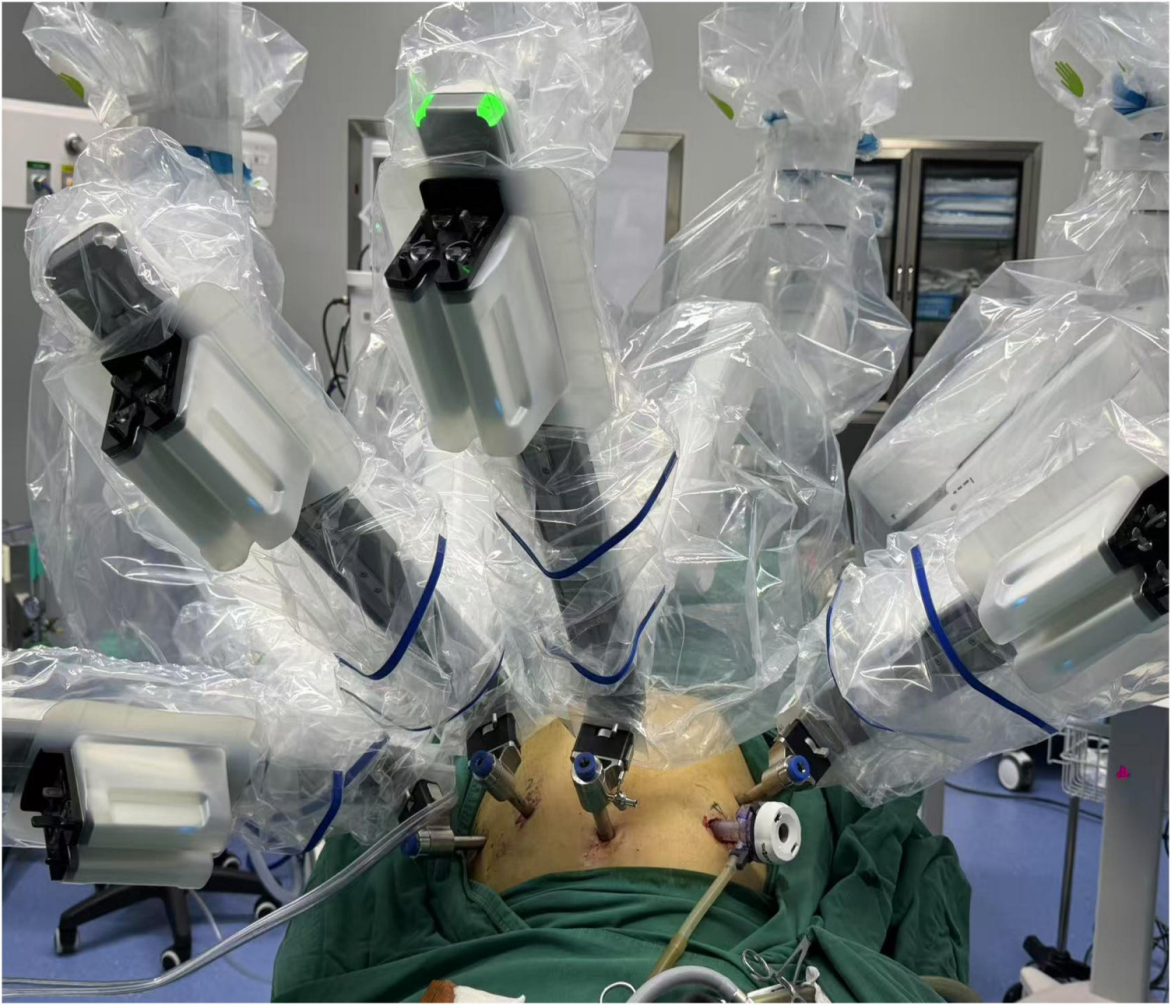
Schematic illustration of trocar placement and port configuration for robot-assisted partial splenectomy using the Toumai Surgical Robot System.

All procedures were performed using the Toumai Surgical Robot System (Shanghai MedBot Group, China). Patients were positioned in the right lateral decubitus position with approximately 30° elevation of the left upper abdomen and a 15° head-up tilt under general anesthesia. The configuration and placement of robotic trocars, along with a 12-mm non-robotic assistant trocar used for suctioning, instrument introduction, and specimen retrieval, are shown in Figure 1.

### 2.4 Surgical procedure

Exploration and Exposure: After entering the abdominal cavity, a thorough exploration was performed to exclude distant lesions or severe adhesions. The splenocolic, splenorenal, and splenophrenic ligaments were divided to fully mobilize the spleen and expose the lesion.

Vascular Control and Parenchymal Transection: Guided by preoperative 3D reconstruction, arterial and venous branches supplying the targeted segment were carefully isolated and temporarily occluded to confirm the ischemic demarcation line. Once the boundary was clearly identified, these vessels were definitively divided using robotic instruments, and parenchymal transection was performed along this line to ensure adequate margins while preserving remnant perfusion.

Hemostasis and Suturing: The transection plane was inspected, superficial bleeding was controlled with bipolar cautery, and 3/4-0 absorbable sutures were used for continuous or interrupted closure. Topical hemostatic agents (oxidized regenerated cellulose, Surgicel®) were applied as necessary.

Specimen Retrieval and Closure: The specimen was placed in an endoscopic retrieval bag and extracted through the assistant port. The abdominal cavity was irrigated and inspected before trocar removal and incision closure.

### 2.5 Postoperative management and follow-up

Postoperative monitoring included vital signs, complete blood count, and drainage volume. A 19 Fr silicone abdominal drain was routinely placed adjacent to the splenic transection surface through the assistant port at the end of the procedure. Oral intake was resumed 24 h after surgery, and drains were removed between postoperative days 5–7 when the following criteria were met: drainage volume <20–30 mL/day, absence of active bleeding, clear or serous drainage, and no clinical or laboratory signs of infection. Pre-discharge imaging (ultrasound or CT) confirmed adequate perfusion of the residual spleen. Ultrasound was used as the routine modality, whereas contrast-enhanced CT was performed when ultrasound findings were inconclusive or when postoperative complications (e.g., hematoma or suspected ischemia) needed to be excluded. Follow-up at 1, 3, and 6 months, and annually thereafter, evaluated hematologic status and recurrence.

### 2.6 Statistical analysis

All analyses were performed using SPSS version 26.0 (IBM Corp., Armonk, NY, USA). Continuous data were expressed as mean ± standard deviation (*x* ± s), and categorical data as counts and percentages [*n* (%)]. As this was a descriptive case series, no inferential statistical tests were conducted.

## 3 Results

### 3.1 Intraoperative findings

Baseline characteristics of the enrolled patients are summarized in Table 1. All nine patients successfully underwent RAPS without conversion to open surgery or major intraoperative complications. The type of resection was determined based on the location of the lesion and the segmental vascular anatomy, with seven cases involving the lower pole and two cases involving the upper pole of the spleen.

**Table 1:** Baseline characteristics of patients with benign splenic lesions (*n* = 9).

No.	Sex	Age (years)	Lesion location	Lesion size (cm)	Main symptom	Preoperative imaging diagnosis	Operative time (min)	Blood loss (mL)	Pathology	Hospital stay (days)
1	F	39	Lower pole	3.1	Incidental finding	Splenic cyst	190	90	Splenic cyst	6
2	F	57	Lower pole	4.7	Left upper abdominal discomfort	Splenic hemangioma	205	60	Splenic hemangioma	7
3	M	52	Lower pole	4.0	Left upper abdominal dull pai	Splenic cyst	180	50	Splenic cyst	6
4	F	40	Upper pole	3.8	Incidental finding	Splenic cyst	195	80	Splenic cyst	5
5	M	68	Lower pole	4.5	Left upper abdominal dull pain	Splenic hemangioma	170	90	Splenic hemangioma	8
6	M	55	Lower pole	3.5	Incidental finding	Splenic hemangioma	160	50	Splenic lymphangioma	7
7	F	37	Upper pole	5.2	Incidental finding	Splenic cyst	165	70	Splenic cyst	6
8	F	42	Lower pole	5.5	Incidental finding	Splenic cyst	185	100	Splenic cyst	7
9	F	51	Lower pole	4.8	Incidental finding	Splenic hemangioma	165	50	Splenic hemangioma	6
Mean ± SD	–	49.0 ± 10.3	–	4.34 ± 0.80	–	–	179.4 ± 15.5	71.1 ± 19.6	–	6.4 ± 0.9

F, female; M, male.

Continuous variables are presented as mean ± standard deviation.

The mean lesion diameter was 4.34 ± 0.8 cm. The mean operative time was 179.4 ± 15.5 min, and the mean estimated blood loss was 71.1 ± 19.6 mL. None of the patients required intraoperative or postoperative blood transfusion. All procedures achieved clear resection margins, and intraoperative assessment—primarily based on direct visual inspection and supplemented by Doppler ultrasonography when necessary—confirmed satisfactory perfusion of the remnant spleen.

### 3.2 Postoperative recovery

All patients recovered uneventfully after surgery. The mean postoperative hospital stay was 6.4 ± 0.9 days. No postoperative hemorrhage, pancreatic fistula, splenic infarction, or severe infection occurred. All patients maintained normal postoperative body temperature, with well-healed incisions and no evidence of drain obstruction or infection.

Before discharge, follow-up ultrasonography or CT imaging demonstrated satisfactory splenic perfusion without areas of non-enhancement or ischemia.

### 3.3 Pathological findings

**Figure 2:**
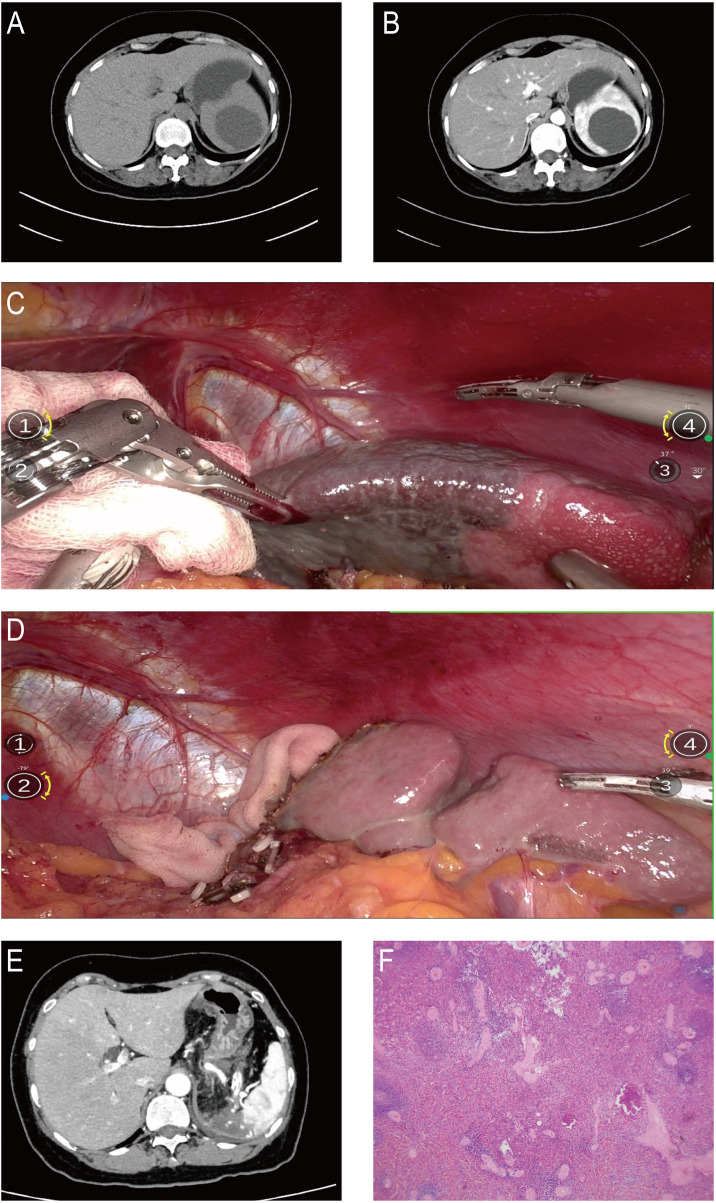
Robot-assisted partial splenectomy for an upper-pole non-parasitic splenic cyst: imaging, intraoperative key steps, and outcomes. **(A,B)** Preoperative upper-abdominal CT in non-contrast and contrast-enhanced phases revealed a well-defined non-parasitic cystic lesion located at the upper pole of the spleen, with no evidence of internal septations or solid components. **(C)** After selective ligation of the upper polar branch of the splenic artery, a distinct ischemic demarcation line appeared between the diseased upper pole and the well-perfused remaining splenic tissue, clearly delineating the resection margin. **(D)** Following resection of the splenic upper pole, the remnant spleen maintained satisfactory perfusion without any signs of ischemia. **(E)** Contrast-enhanced CT performed on postoperative day 7 demonstrated good perfusion of the residual spleen, without hematoma or fluid collection. **(F)** Histopathological examination confirmed the diagnosis of a splenic cyst.

Histopathological examination confirmed that all lesions were benign. The diagnoses included splenic hemangioma (*n* = 3), non-parasitic splenic cyst (*n* = 5), and splenic lymphangioma (*n* = 1). Representative preoperative imaging, intraoperative view, and postoperative histopathological findings of a typical case are shown in Figure 2.

### 3.4 Postoperative splenic function and follow-up

Complete blood counts performed on postoperative day 7 revealed a mean platelet count of 258 ± 47 × 10⁹/L, slightly higher than the preoperative level (241 ± 52 × 10⁹/L), but still within the normal range. White blood cell and red blood cell counts showed no significant abnormalities. No cases of hypersplenism or hematologic dysfunction were observed, indicating preserved splenic function in all patients.

During follow-up, all patients remained in good general condition. Imaging examinations (ultrasound or contrast-enhanced CT) revealed no evidence of lesion recurrence, perfusion defects in the remnant spleen, or new lesions. No delayed splenic infarction, postoperative cyst recurrence, or infectious complications were detected throughout the follow-up period.

## 4 Discussion

The spleen plays a crucial role in both the immune and hematopoietic systems, being responsible for clearing senescent erythrocytes, synthesizing immunoglobulins, phagocytosing bacteria, and regulating immune responses (1–3). Historically, limited understanding of splenic function led clinicians to favor total splenectomy for treating splenic space-occupying lesions. Although this procedure achieves complete removal of the lesion, it is associated with a decline in immune function and persistent thrombocytosis. In severe cases, patients may develop overwhelming post-splenectomy infection, a life-threatening condition with extremely high mortality (10–12). Moreover, long-term follow-up studies have shown that total splenectomy increases the risk of thromboembolic events, atherosclerosis, and metabolic disorders (13–15).

Consequently, preserving functional splenic tissue whenever feasible—while ensuring complete removal of the lesion—has become a widely accepted principle in modern splenic surgery. Studies have demonstrated that retaining even a portion of normal splenic tissue is sufficient to maintain basic immune competence and significantly reduce the incidence of infection and hematologic abnormalities. These findings provide solid theoretical and clinical justification for PS as an effective spleen-preserving surgical strategy (6, 9, 16).

The development of laparoscopic technology has enabled PS to evolve from open to minimally invasive approaches. LPS offers the advantages of reduced trauma, less pain, and faster recovery, in line with the principles of enhanced recovery after surgery. Numerous studies have reported that LPS provides superior postoperative recovery and lower complication rates compared with open surgery (17–20). However, due to the spleen's rich vascularization, complex hilar anatomy, and friable parenchyma, LPS remains technically demanding. The limitations of two-dimensional vision and restricted instrument motion can hinder accurate identification of segmental blood supply, determination of transection planes, and secure hemostasis and suturing. These challenges are further amplified when the lesion is located near the splenic hilum or deep parenchyma, where restricted space and limited maneuverability increase the risks of bleeding, parenchymal leakage, and impaired remnant perfusion. These factors have constrained the widespread adoption of LPS for complex splenic lesions.

The advent of robot-assisted surgical systems has introduced new opportunities for spleen-preserving surgery. The robotic platform provides a high-definition 3D view, tremor filtration, and articulated instruments with multiple degrees of freedom, enabling precise dissection and suturing in confined anatomical spaces. These features substantially enhance operative precision and safety. In the present study, all cases of PS were completed using a robotic system with excellent visualization, accurate vascular identification, and smooth suturing. The mean intraoperative blood loss was less than 100 mL, and no conversions to open surgery or major bleeding events occurred, underscoring the safety and controllability of robotic technology in minimally invasive spleen-preserving surgery.

Another technical highlight of this study is the use of preoperative 3D reconstruction to evaluate the spatial relationship between the lesion and splenic vasculature. The splenic hilum is characterized by considerable vascular complexity and individual variation, which are often difficult to assess with conventional two-dimensional imaging. By integrating contrast-enhanced CT data, 3D reconstruction allows intuitive visualization of the main and segmental branches of the splenic artery and vein, clearly delineating the vascular territories associated with the lesion. This enables the surgeon to develop a precise preoperative resection plan. With robotic assistance, targeted vascular branches can be accurately isolated and divided according to the 3D roadmap, ensuring complete lesion removal while maximizing perfusion of the remnant spleen and reducing intraoperative risks. Postoperative imaging in all patients demonstrated adequate splenic perfusion without focal infarction, confirming the reliability and safety of combining preoperative 3D planning with robotic technology.

The findings of this study demonstrate that RAPS is both feasible and safe for the treatment of BSLs. All procedures were successfully completed without conversion to open surgery, and no major postoperative complications such as pancreatic fistula, splenic infarction, or infection were observed. The mean operative time, mean blood loss, and mean hospital stay were comparable to previously reported data. All pathological diagnoses were benign, and no recurrence or splenic dysfunction was detected during follow-up, indicating that RAPS achieves radical resection while preserving splenic function.

Beyond its technical precision, robotic surgery also offers advantages in the learning curve and team coordination. Evidence suggests that the robotic system shortens the learning curve, enabling surgeons to master complex anatomical procedures within a shorter period and improving procedural consistency. Furthermore, the system's tremor filtration and precise instrument control provide stable support for operations involving delicate vascular structures, thereby minimizing intraoperative complications. In our experience, operative time gradually decreased after the initial learning phase, which encompassed approximately the first five cases, reflecting the reproducibility and clinical scalability of the robotic platform.

Nevertheless, several limitations should be acknowledged. This was a single-center retrospective study with a small sample size and limited follow-up duration. Additionally, direct comparative data with LPS were not available, precluding definitive conclusions regarding differences in postoperative immune recovery or long-term outcomes. Moreover, the high cost and maintenance requirements of robotic systems may restrict their widespread implementation in resource-limited settings. Future multicenter prospective randomized controlled trials are warranted to further validate the long-term efficacy and immunologic advantages of RAPS. Integration of emerging technologies such as artificial intelligence–assisted imaging and preoperative vascular modeling may further enhance precision and individualization in spleen-preserving surgery.

In summary, RAPS combined with preoperative 3D reconstruction enables precise preoperative planning and meticulous intraoperative execution, achieving the dual goals of complete lesion resection and maximal preservation of splenic function. This approach exemplifies the evolution of precision and minimally invasive surgery, offering a safe, feasible, and promising technique for the management of BSLs.

## 5 Conclusion

RAPS is a safe and feasible procedure for the management of BSLs. It enables precise resection of the lesion while preserving sufficient functional splenic tissue to maintain physiological immune function. With the accumulation of surgical experience and the wider availability of robotic systems, RAPS is expected to play an increasingly significant role in the field of minimally invasive splenic surgery.

## Data Availability

The original contributions presented in the study are included in the article/Supplementary Material, further inquiries can be directed to the corresponding authors.
